# C-856-05. Improving Burn Care: Insights from Social Vulnerability Index Themes

**DOI:** 10.1093/jbcr/irag033.097

**Published:** 2026-04-06

**Authors:** Meredith Hanrahan, Lori Rhodes, Jeffrey Anderson, Jingwei Wu

**Affiliations:** Temple University Hospital, Philadelphia, PA; Temple University Hospital, Philadelphia, PA; Medical College of Wisconsin, Milwaukee, WI; Temple University, Philadelphia, PA

## Abstract

**Introduction:**

The CDC's Social Vulnerability Index (SVI) quantifies a community's predisposition to inadequate disaster recovery and health disparities. This study aimed to determine the effectiveness of individual SVI themes in predicting burn characteristics and critical outcomes among patients treated at a high-volume, urban burn center.

**Methods:**

This retrospective cohort study from 2020 to 2023 analyzed adult burn patients admitted to an American Burn Association-verified urban academic burn center. Patients were categorized into low-risk (1st-3rd quartiles) and high-risk (4th quartile) groups based on social vulnerability across four SVI themes: socioeconomic status (SES), household characteristics (HC), racial & ethnic minority status (RE), and housing type & transportation (HTT), derived from patient addresses. Univariate analysis compared burn characteristics and multivariate analysis, adjusting for age, sex, ethnicity, diabetes, insurance, housing status, inhalation injury, delayed presentation, and %TBSA, compared patient outcomes between high and low-risk groups for each theme.

**Results:**

Of the 699 subjects, the median age was 52 years (IQR 38-65 years) and 62.5% of subjects were male. Median %TBSA was 3 (IQR 1-7). Subjects in the high-risk group in each theme were more likely to be of African American or Hispanic background, have Medicaid insurance, and be unemployed (*p*<.01). High-risk SES, HC, and RE groups were more likely to sustain flame burns compared to other mechanisms (*p*<.01), while high-risk SES and HC were associated with delayed patient presentation (*p*<.01). Both high risk SES and high risk HTT were predictors of wound infection when adjusting for confounders (OR: 1.50, 95% CI: 1.07-2.08; OR: 1.48, 95% CI: 1.03-2.13). No significant differences were found in discharge destination, unplanned readmission, follow-up length of stay, or death in adjusted models.

**Conclusions:**

High-risk social vulnerability, particularly within the SES, HC, and RE themes, is significantly associated with specific burn characteristics including flame mechanism and delayed presentation as well as demographic disparities. High-risk SES and HTT independently predict an increased incidence of wound infection.

**Applicability of Research to Practice:**

Results of the study highlight the utility of SVI themes in identifying populations vulnerable to distinct burn injury profiles and complications, underscoring opportunities for targeted public health interventions and tailored clinical strategies to improve burn care outcomes.

**Funding for the study:**

N/A.

